# Animal Intelligence

**Published:** 1888-05

**Authors:** 


### ANIMAL INTELLIGENCE.

A New York paper speaks of a couple of orioles that have built a nest in a tree at Central Park. In order that no animal able to climb a tree might reach their nest, they built their habitation at the extreme end of a light branch, and when the work was half done they saw that the little house was bending the branch so far towards the earth that when the house was full of young ones it would be so near the ground that dogs and cats might reach it.

Here is where the reasoning power showed itself. The birds sat on the branch studying their house for a time, and then flew off in search of a string. They found some twine in the Park, and with it they united the too elastic boughs to a sturdy branch overhead, resuming their nest-building after the string had been firmly woven. The branch can now sag only to the limits of this cleverly contrived stay, and will not bend far enough toward the earth to expose their young to danger from cats or other predatory animals. There are brains in that engineering operation.

